# Interdisciplinary and Collaborative Approaches Needed to Determine Impact of COVID-19 on Older Adults and Aging: CAG/ACG and *CJA*/*RCV* Joint Statement

**DOI:** 10.1017/S0714980820000203

**Published:** 2020-05-15

**Authors:** Brad A. Meisner, Veronique Boscart, Pierrette Gaudreau, Paul Stolee, Patricia Ebert, Michelle Heyer, Laura Kadowaki, Christine Kelly, Mélanie Levasseur, Ariane S. Massie, Verena Menec, Laura Middleton, Linda Sheiban Taucar, Wendy Loken Thornton, Catherine Tong, Deborah K. van den Hoonaard, Kimberley Wilson

**Affiliations:** 1 Board of Directors, Canadian Association on Gerontology; 2 Editorial Board, Canadian Journal on Aging; 3 School of Kinesiology & Health Science, York University; 4 School of Health & Life Sciences/Schlegel Centre for Advancing Seniors Care, Conestoga College; 5 Department of Medicine, Université de Montreal; 6 School of Public Health & Health Systems, University of Waterloo; 7 Seniors Health Services, Alberta Health Services/Department of Psychology, University of Calgary; 8 Student Connection, Canadian Association on Gerontology; 9 Department of Gerontology, Simon Fraser University; 10 Department of Community Health Sciences, University of Manitoba; 11 School of Rehabilitation, Université de Sherbrooke; 12 Department of Kinesiology, University of Waterloo; 13 Department of Psychology, Simon Fraser University; 14 Department of Gerontology, St. Thomas University; 15 Department of Family Relations & Applied Nutrition, University of Guelph

**Keywords:** vieillissement, coronavirus, relations interprofessionnelles, communication savante, recherche interdisciplinaire, collaboration intersectorielle, aging, coronavirus, interprofessional relations, scholarly communication, interdisciplinary research, intersectoral collaboration

## Abstract

The COVID-19 pandemic and subsequent state of public emergency have significantly affected older adults in Canada and worldwide. It is imperative that the gerontological response be efficient and effective. In this statement, the board members of the Canadian Association on Gerontology/L’Association canadienne de gérontologie (CAG/ACG) and the *Canadian Journal on Aging*/*La revue canadienne du vieillissement* (*CJA*/*RCV*) acknowledge the contributions of CAG/ACG members and *CJA*/*RCV* readers. We also profile the complex ways that COVID-19 is affecting older adults, from individual to population levels, and advocate for the adoption of multidisciplinary collaborative teams to bring together different perspectives, areas of expertise, and methods of evaluation in the COVID-19 response.

## RÉSUMÉ

La pandémie, et l’état d’urgence face à la pandémie, de COVID-19 a des répercussions significatives pour les personnes âgées au Canada et à travers le monde. Il est donc impératif que la réponse du domaine de gérontologie soit efficace. Dans cette déclaration, les membres du conseil administratif de l’Association canadienne de gérontologie/Canadian Association on Gerontology (ACG/CAG) et *La revue canadienne du vieillissement*/*Canadian Journal on Aging* (*RCV*/*CJA*) reconnaissent la contribution des membres de l’ACG/CAG et des lecteurs de la *RCV*/*CJA.* Nous présentons également comment COVID-19 affectent de façon complexes les personnes âgées, de l’individu à la population, et préconisons l’adoption d’équipes pluridisciplinaires et collaboratives pour rassembler des perspectives variées, différents domaines de compétences, et méthodes d’évaluation dans la réponse à la pandémie COVID-19.

## Introduction

The COVID-19 pandemic has resulted in numerous unprecedented changes across Canada. Government restrictions on mobility and closures of non-essential workplaces, recommendations regarding physical distancing and isolation, the virtualization of work and schooling, and the increased demand on essential health care services have dramatically altered daily life for Canadians and citizens abroad. Although COVID-19 affects us all, arguably, older adults are affected the most by this pandemic, which has shed a light on previously acknowledged and unacknowledged challenges facing this part of the population. There exists an urgent need for effective responses to COVID-19 concerning its influences on older adults in Canada and around the world. As leading national scientific, educational, and dissemination organizations on matters related to older adults and aging in Canada, the Canadian Association on Gerontology/L’Association canadienne de gérontologie (CAG/ACG) and the *Canadian Journal on Aging*/*La Revue canadienne du vieillissement* (*CJA*/*RCV*) have prepared this statement with two key objectives in mind.

First, we want to acknowledge our CAG/ACG members and *CJA*/*RCV* readers. *Thank you.* Representing a wide-ranging and active mix of researchers, professionals, organizations, students, caregivers, and older adults across Canada, many CAG/ACG members and *CJA*/*RCV* readers are working directly or indirectly with older adults during this pandemic. This work, which is more important now than perhaps ever before, is taking place under challenging conditions within and across multiple sectors. We want to express our collective appreciation for your contributions to academia and higher education, health care practice and administration, community care and development, as well as policy and governance during the COVID-19 pandemic. As you continue your work, we will provide you with information and evidence as well as opportunities for engagement, discussion, and collaboration via email, social media, websites, webinars, and forthcoming published papers on COVID-19.

Second, we want to recognize the complex and multifaceted ways that COVID-19 is affecting older adults from individual to population levels – from SARS-CoV-2 as an infective agent to the societal and historical contexts of the pandemic. No single discipline will be able to discern why, how, and how much older adults are and will be impacted by COVID-19. As such, we strongly encourage the adoption of interdisciplinary approaches in the response to COVID-19 because of the value added when connections between and across disciplines are made. To illustrate the need for interdisciplinary perspectives, in the following sections, we identify and highlight just some examples of important issues and opportunities that exist pertaining to COVID-19 in gerontology. These points are meant to stimulate reflection as well as generate research questions and agendas pertaining to COVID-19, older adults, and aging.

## Challenges and Opportunities for Supporting Psychological Health and Well-Being

### Patricia Ebert and Wendy Loken Thornton

There are unique psychological challenges that many older adults experience amid COVID-19 including increased social isolation and the emergence or exacerbation of mental health issues, such as stress, substance use, anxiety, and depression. Although recent polls find that older adults are less likely than younger adults to report worry or stress related to COVID-19 (Angus Reed Institute, 2020; Panchal et al., 2020), this finding must be considered in the context of the pre-existing social and mental health status of older Canadians. For example, approximately 15 per cent of older Canadians do not have close friends (Turcotte, 2015), 25 per cent of those 85 years of age and greater experience loneliness (Gilmour, 2012), up to 15 per cent of older adults experience significant symptoms of depression (Canadian Psychological Association, 2015), and adults 55 years of age and greater have the second-highest prevalence rate of mood and anxiety disorders (McRae, O’Donnell, Loukine, Rancourt, & Pelletier, 2016). Reactive or pre-existing mental disorders may be exacerbated among older adults by the intense fear of COVID-19 infection (i.e., coronophobia) (Asmundson & Taylor, 2020), and those with pre-existing physical conditions (e.g., COPD, obesity) may experience enhanced existential distress and concerns about death and dying.

Another issue is the exacerbation of older adult, caregiver, and care provider stress as many homecare, community, and dementia supports (e.g., day programming) are reduced, cancelled, or modified to be online. The increased reliance on virtual or internet-based services may be a barrier, however. Despite evidence that older adults who use video chat technology have lower rates of depression (Teo, Markwardt, & Hinton, 2019), older adults are also most likely to be without internet access (Statistics Canada, 2019) or the knowledge, skills, and confidence necessary to use emerging technologies effectively, particularly if they are experiencing cognitive decline (Wild et al., 2012). This relative lack of technological efficacy may also contribute to the susceptibility to financial scams and fraud, which have increased since the pandemic, further victimizing those who are lonely and socially isolated (Canadian Anti-Fraud Centre, 2020). Therefore, practical solutions are needed to improve mental health and alleviate isolation and victimization of older adults.

Further, there are concerns for potential cognitive decline secondary to otherwise life-saving COVID-19 interventions. Many older and sicker patients require ventilator assistance for considerable periods, which may lead to an aftermath of health and cognitive disabilities resulting from post-intensive care syndrome (Stam, Stucki, & Bickenbach, 2020). These include depression and impairments in memory, attention, and other neuropsychological functions (Pandharidpande et al., 2013; Stam et al., 2020). These cognitive challenges will require training initiatives for those working in psychological and rehabilitation capacities with older post–COVID-19 patients. Psychologists should also focus on developing empirically based strategies for assisting older patients with emerging and lingering mental health issues, including those resulting in cognitive challenges (Gallagher, McLeod, & McMillan, 2019). Developing scales to assess the cognitive and neuropsychiatric impact of COVID-19 infection-related control measures (e.g., physical distancing) are needed. Moving forward, cognitive and neuropsychological screening, assessment, and management will need to be an ongoing part of psychological care for older adults.

## Challenges and Opportunities for Supporting Health Behaviours and Well-Being

### Laura Middleton

The rapid behavioural change required by the COVID-19 public health response will likely lead to short- and long-term adverse health consequences. Older Canadians face restricted community mobility, loss of social support, and reduced access to services and goods. Community-based programs that support social, mental, and physical well-being are shut down or severely restricted. It is likely that daily sedentary behaviour, which is associated with increased risk of cardiovascular and all-cause mortality, will increase (Biswas et al., 2015). At-home exercise is more challenging for many people because there is little social support, encouragement, and accountability, which are key facilitators of physical activity (Schutzer & Graves, 2004). Also, recommendations to reduce or avoid shopping may restrict access to healthy food. Social isolation and loneliness, because of physical distancing, are strong predictors of morbidity and mortality in later life, even though the pathways are complex (Freedman & Nicolle, 2020). As a result of COVID-19, modifiable lifestyle risk factors for chronic disease, functional disability, and mortality are likely to be accentuated.

More optimistically, novel solutions are emerging from individuals, organizations, and businesses to support the health and well-being of older adults in communities. There are many examples of neighbours, students, and younger people supporting older adults in this time. For example, “Chatting to Wellness” is a student-run, non-profit organization that provides social connection to older adults, which continues via telephone during the COVID-19 pandemic (Jackson, 2020). Community organizations and businesses such as the YMCA, the Alzheimer Society, and local yoga studios are turning to online programming, emailed newsletters and resources, and web-based programs and support groups. Although older adults are typically the lowest adopters of technologies such as computers and smartphones (Vogels, 2019), the COVID-19 pandemic may be the impetus among older adults to adopt these technologies (Finn, 2020), even among those with cognitive impairment, especially when supported by family members (Wild et al., 2012). However, lack of equipment and internet access, particularly in rural and remote areas, are challenging barriers to overcome. Consequently, the benefits of technology may be restricted only to the two thirds of older adults who already use the internet (Anderson & Perrin, 2017) and the two fifths who already have smartphones (Vogels, 2019). It will also be important to consider how the lessons learned and resources developed in response to the threat that COVID-19 has on health behaviours and its associated outcomes could underpin the establishment and sustainability of programs and services for physically or socially isolated older Canadians in the future.

## Experiences and Challenges in Long-Term Care

### Veronique Boscart, Michelle Heyer, and Linda Sheiban Taucar

Older adults, especially those in long-term care (LTC) homes, are severely affected by the spread of COVID-19. COVID-19 cases are more severe in individuals who experience multiple morbidities and weaker immune systems, allowing viral infections to progress much more quickly (Rothan & Byrareddy, 2020). Residents and those who care for them are becoming infected, leading to an increased mortality rate among the most vulnerable. Deaths resulting from COVID-19 in LTC are estimated to represent approximately 63 per cent of overall COVID-19 deaths in Canada (Hsu & Lane, 2020). In Ontario alone, more than 150 LTC homes have or have had outbreaks of COVID-19 (Government of Ontario, 2020). These outbreaks affect not only the overall physical health of individuals, but also the social aspects of people’s lives. LTC homes now restrict visitors, leaving some residents socially isolated. Due to cognitive changes, some residents may not understand why their loved ones are unable to visit them, leading to anxiety and feelings of loneliness. The Government of Canada (2020a) is making efforts to identify and prevent care providers from working in more than one location, which is expected to assist in slowing the spread of COVID-19. Further, in some LTC homes, there exists a shortage of care providers. For those working in homes with limited staff, it is expected that there will be increased levels of burnout. Proper resources will be needed to ensure those who are negatively affected by COVID-19 – physically, psychologically, behaviourally, and socially – are able to seek and receive appropriate supports.

## Not the “Great Equalizer”: Precariousness During the Pandemic

### Catherine Tong

Despite some notoriety in popular culture, COVID-19 is not the “great equalizer” (Owoseje, 2020). Not all persons are experiencing this pandemic with the same set of advantages and resources. Although the present focus, justifiably, has been on LTC, the ongoing pandemic also highlights the precariousness of older adults in receipt of home care and other community-based supports. Researchers have long sounded the alarm that home support workers are providing care in unregulated private residences, up to a dozen per day, and largely function as lone operators (Craven, Byrne, Sims-Gould, & Martin-Matthews, 2012; Doran et al., 2009; Macdonald & McLean, 2018). Given the precarious nature of their work (Zagrodney & Saks, 2017), many personal support workers work both in LTC and home care, although some provinces have suspended this practice in the response to COVID-19 (DeClerq, 2020; Eagland, 2020).

Further, family caregivers across the country are being impacted by COVID-19. For those committed to patient and family engagement (Change Foundation, 2016), it is distressing to see that caregivers, previously deemed “care partners”, are no longer allowed into many of the facilities where their family members are receiving acute care, navigating a health care system that has rapidly changed, and, in the worst-case scenario, taking their last breath. For many family caregivers, these experiences are occurring while they simultaneously balance myriad roles and responsibilities (e.g., caregiver, employee, parent, etc.) – all at a distance. Thus, family caregivers are facing an even greater level of precarity, with new and unique demands on account of COVID-19, which will have long-term ramifications for individual family caregivers and the broader health care system.

Another form of precariousness exacerbated by the pandemic is the profound and potentially disproportionate impact on those who identify as visible/ethnocultural minorities. Nearly one in seven older Canadians is a visible minority (Statistics Canada, 2016) with heightened vulnerabilities to COVID-19 due to their age, ethnicity, and other intersections (Hankivsky, 2011). Although some cities, such as Toronto, have opted to collect ethnicity data in their COVID-19 tracking (Mojtehedzadeh, 2020), there are no plans to do so federally (Nasser, 2020). To understand the impact of COVID-19 on older visible/ethnocultural minorities, a range of methodological approaches and culturally appropriate techniques will be required (Liamputtong, 2010).

Collectively, the research community will need to meaningfully engage with groups who are facing this pandemic from a precarious position. Although it is crucial to identify individuals and groups who face systemic and perpetuated disadvantages, it is equally important to identify and highlight the resiliency and strengths within these communities as well. A strengths-based focus (Moyle, Parker, & Bramble, 2014), collaborative research (Marlett & Emes, 2010), and authentic co-design (Donetto, Tsianakas, & Robert, 2014) are all approaches that will allow our gerontological community to understand and respond to this pandemic while being especially mindful of those experiencing exacerbated precariousness due to COVID-19.

## Call to Remember the Social and Cultural Aspects of Aging and Health

### Christine Kelly

COVID-19 is not an equitable disease. Of course, older people, people with disabilities, and people with underlying health conditions are at higher risk of contracting more serious forms of COVID-19. However, it is also emerging that COVID-19 disproportionately affects people of colour (Ormiston, 2020). This evolving reality highlights how systemic structures of exclusion and marginalization result in lived experiences of heightened risk and oppression during the global pandemic. Therefore, we must specifically consider how ageism, ableism, racism, and other forms of systemic oppression are shaping public discourses around COVID-19. It is essential to consider these broader social issues when studying and responding to COVID-19. The social lens cannot be removed from the physiological and political responses to this situation.

At present, the research industry and media are emphasizing clinical trials and intervention-based research on COVID-19. However, even the most medically oriented area of work should also take into account social factors through the inclusion of social scientists and humanists, and through the use of relevant and critical theoretical tools such as intersectionality (Rice, Harrison, & Friedman, 2019), health equity (Marmot & Allen, 2014), and precarity (Grenier, Lloyd, & Phillipson, 2017). These are just some of many potential starting points for understanding the broader social structures that influence the COVID-19 contexts for diverse older people. In addition to adding a social lens to medical research, there is a need for research that employs qualitative methods that can gather perceptions and accounts of varied lived experiences during this time. Such research will require creative adaptation to adhere to physical distancing guidelines.

Finally, cultural gerontology, as a broader application of the humanities in the study of aging, has made significant contributions to our understanding of age and aging (Twigg & Martin, 2015). Perhaps most importantly, this type of work can generate new theoretical orientations and help analyze in-depth artistic representations of this time as they will emerge in music, literature, film, and other media. Many of us will notice a surge in creative arts – poetry, dance, music, visual – all inspired by this unusual moment in history. We are turning to the arts as a form of comfort, demonstrating that creative arts, and our analysis of them, are not supplementary but vital to our processing of the current reality. During this extraordinary time, researchers, practitioners, and others are urged not to forget the social and cultural aspects of health and aging.

## The Importance of Older Adults’ Perspectives on Life During the Pandemic

### Deborah K. van den Hoonaard

Although COVID-19 seems to be primarily an issue related to health, there is need for important research that helps us understand the social meaning and lived experience of being an older adult in this pandemic. We should interrogate the way the older population has been described in the media and by politicians and health experts. Describing the older population as “the elderly” makes all older people seem to be alike even though they comprise a very diverse group (Applewhite, 2016; Gullette, 2004). The re-emergence of the phrase “grey tsunami” is troubling. Seeing the older population as fully human requires upending the hierarchy of credibility (Becker, 1967; van den Hoonaard, 2018), which prioritizes the opinions of “experts” over the perspectives of older people. It will be important for us to understand how the everyday lives of older adults changed during this period. Sociologists and narrative scholars can develop a “collective story” that can “give voice to those whose narratives have been excluded from the public domain and civic discourse, thereby making collective identity, and collective solutions possible” (Richardson, 1990, p. 28). As we go forward, it will be important to conduct inductive, qualitative research that provides the perspectives of older people about the impact of COVID-19 on their lives. Our tendency as gerontologists to see older people as vulnerable is challenged when we take notice of their points of view, creativity, and resilience, and when we take a more holistic view that recognizes that there is more than health to think about even during a pandemic.

## Critical Views on Aging Amid COVID-19: A Call for More Gerontological Expertise

### Brad A. Meisner, Ariane S. Massie, and Laura Kadowaki

Canadians are being inundated with local and global COVID-19 public health campaigns. An examination of how these campaigns have contributed to the (mis)understanding of aging and older adults is warranted. It is now popular knowledge that the risks for adverse medical outcomes is significantly higher for older adults than it is for children, youth, and younger adults. Both the Government of Canada (2020b) and the World Health Organization (2020) identify older adults as the group most likely to experience severe illness, hospitalization, intensive care, and death because of COVID-19. However, the use and representation of chronological age in these campaign messages can be critiqued in several ways. First, age is categorized using cut-off points as the “older adult” group is defined as those aged either 60 or 65 years and greater (World Health Organization, 2020; Government of Canada, 2020b; respectively).

From a lifespan perspective, the use of chronological age cut-off points is arbitrary and should be avoided as it falsely represents older adults as a homogenous group (Ayalon et al., 2020). Such cut-off points fail to account for the biological, psychological, social, and ecological individual differences and diversities that exist among older adults, especially for an age classification that spans many decades. Second, the messages disseminated about COVID-19 risk are being attributed to chronological age when other factors appear to be more robustly associated to this risk than age (Montero-Odasso et al., 2020). For example, although hospitalization rates are highest among older adults, a clear majority of hospitalized patients have underlying health conditions – regardless of age (Garg et al., 2020). Third, the use of arbitrary age cut-offs and the generalized (mis)attribution of COVID-19 risk to aging has inadvertently reinforced and intensified negative age stereotypes, prejudice, and discrimination that will need to be addressed (Ayalon et al., 2020; Brooke & Jackson, 2020; Meisner, 2020). Even before the pandemic, interventions aiming to reduce ageism were essential and thus should be developed further (Burnes et al., 2019).

To address ageism and to discern the facts and fictions of COVID-19 and aging more broadly, there is a need for a greater number and involvement of professionals with gerontological training and expertise. Currently, there is a lack of undergraduate education and a limited number of graduate programs on gerontology in Canada (Wister, Kadowaki, & Mitchell, 2016). Higher education institutions may need to reimagine their approach to student recruitment for these programs. Students’ perceptions of working with older adults are mostly negative, which deters them from pursuing higher education on aging or considering a career working with older adults (Algoso, Peters, Ramjan, & East, 2016). Given the intensification of ageist discourse associated with COVID-19 (Meisner, 2020), this trend is unlikely to change, much less improve. Once in gerontology programs, students need effective learning opportunities. Education on, and quality interactions with, older adults through experiential learning reduces harmful age biases (Obhi & Woodhead, 2016) and increases one’s interest in working with older adults (Allen, Kelly, Brooks, & Barnard, 2014). These learning experiences must be modified (e.g., via virtual interactions) to respond to the current and potential future public health emergencies. This crisis provides an incentive to invest more in gerontology education and an opportunity to redesign curricula to address important issues, which will benefit the aging population and society long after this pandemic is over.

## COVID-19- and Aging-Related Research in Quebec

### Mélanie Levasseur and Pierrette Gaudreau

Quebec has a distinct COVID-19 profile in Canada. As of May 7, 54 per cent of COVID-19 cases and 60 per cent of deaths in Canada occurred in Quebec (Government of Canada, 2020c; l’Institut national de santé publique du Québec, 2020). Considered the epicentre of COVID-19 in Canada, the city of Montreal alone currently has 17,918 confirmed cases and 1,666 deaths (Government of Québec, 2020). As in other provinces and countries, deaths are overrepresented in people aged 80 years and greater. In response to these staggering numbers and trends, the Quebec Network for Research on Aging/Le Réseau québécois de recherche sur le vieillissement launched a COVID-19 research grant competition with financial support from the Quebec Research Fund–Health/le Fonds de recherche du Québec–Santé. Three COVID-19 research priority themes were identified from this provincial competition, which spans biological, psychological, social, and societal perspectives.

The first theme includes projects that aim to improve the quality of life and experiences of older adults in different physical and social living environments amid COVID-19 (e.g., LTC, community settings), including older adults with physical impairment or psychological distress and those facing social isolation or lacking caregiver support. The second theme includes projects that aim to develop, implement, and evaluate new tools and strategies to preserve the health status, functional mobility, and quality of life of older adults affected by COVID-19. The third theme includes projects that aim to organize and evaluate the accessibility of health care systems and strategies used by care and community organizations to adapt the provision of support services to older adults and caregivers in times of physical distancing and social isolation. The research funded within these themes represents part of Quebec’s response to COVID-19 and how it affects aging and older adults. The findings from these projects will contribute to addressing the issues and identifying the opportunities for promoting health and quality of life among older adults living in a range of environments with a range of health conditions. The expected outcomes will provide a collective understanding of the impacts of COVID-19 and lead us to be better prepared for future public emergencies.

## COVID-19 and the Canadian Longitudinal Study on Aging

### Verena Menec

The Canadian Longitudinal Study on Aging (CLSA) is uniquely positioned to provide a data platform for interdisciplinary research on the complexities and consequences of COVID-19 on older adults and aging in Canada. The CLSA is a national strategic initiative of the Canadian Institutes for Health Research designed to understand diverse topics and issues related to aging (Kirkland et al., 2015; Raina et al., 2009, 2019). Launched in 2010, the study included 51,339 Canadians aged 45 to 85 years at baseline. Participants completed their first follow-up in the years 2015 through 2018 with the second follow-up currently underway. Interview, physical assessment, and biomarker data are available for researchers via a data access application process (see https://www.clsa-elcv.ca). In response to the current pandemic, the CLSA team launched a COVID-19 sub-study in April 2020, conducting web-based and telephone interviews. These interviews collect information on the symptoms, risk factors, health care use, and many other important items associated with COVID-19, including questions about physical distancing and its influence on mental health. The baseline COVID-19 questionnaire will be followed by weekly, biweekly, and monthly questionnaires for a six-month period. COVID-19-specific data will be available for use in combination with the information already available in the CLSA. The CLSA will serve as a very rich data source to identify and address some of the key issues and opportunities highlighted in this statement and others.

## The Global Natural Experiment

### Paul Stolee

COVID-19 is a global pandemic and, while nearly every country in the world has reported cases of the virus, there are great differences in how jurisdictions have responded (Hale, Petherick, Phillips, & Webster, 2020). Responses to the pandemic are driven in part by data and mathematical models, but also by political contexts (Wenham, 2020). Most countries have implemented measures that enforce physical distancing. However, Sweden has controversially resisted a “lockdown” approach (Ward, 2020), and the federal response and variations in regional responses in the United States are widely criticized (Lasry et al., 2020; Villarreal, 2020). Conversely, some leaders, both internationally and within Canada, are celebrated for responding more effectively. Internationally, New Zealand is one example (Friedman, 2020). Within Canada, British Columbia’s timely public health measures have been more successful in “flattening the curve” (Mason, 2020). International and national variations in public health and societal responses to COVID-19 are described as a “global natural experiment” (Thomson, 2020). Varying levels of public jurisdictions are described as “laboratories” (Karch, 2007) because they have the authority to trial different programs and policies as interventions.

Canada’s federal system of government allows for public health and policy responses to COVID-19 to vary nationally, provincially, and territorially, as well as at the municipal level (Meekison, 1977; Simeon, 2006). All jurisdictions report cases of COVID-19 (with the exception of Nunavut at this time) and have implemented stringent measures related to physical distancing. However, again, their implementation, and that of other public health responses, are variable and temporal. For instance, there was an emphasis on the preparation of the acute care system, possibly at the expense of residential and community care. In early March 2020, public policy experts were calling for hospital beds to be freed up by discharging patients to homecare or LTC (Forest & Sutherland, 2020). In mid-March 2020, the Ontario government announced that it was “taking all the necessary precautions to ensure loved ones in Ontario’s LTC homes are safe and secure” (Crawley, 2020) with no deaths yet reported in Ontario LTC homes. But by mid-April 2020, nearly half of Canada’s COVID-19 deaths had links to LTC homes (Aiello, 2020), and on April 22 the Ontario government asked for assistance at five Ontario LTC homes from the Canadian Armed Forces (Crawley, 2020). Also, in mid-April 2020, the federal government issued public health guidance for LTC homes (Public Health Agency of Canada, 2020), and the Ontario government issued a COVID-19 action plan for LTC (Ontario Ministry of Long-Term Care, 2020).

No one asked for or wants the learning opportunity presented by the COVID-19 pandemic. Nonetheless, it has shone a harsh light on deficiencies in our society’s support of older adults. The cost of this neglect is being measured by the alarming infection and mortality rates of older adults, mostly tragically in a private LTC home in Dorval, QC (Feith, 2020). It was not inevitable that this be so, as early responses focused on LTC homes were successful in avoiding outbreaks in some regions (Howlett, 2020). It is imperative that we take advantage of the “natural experiment” presented by differing policy and public health responses taking place in the multiple “laboratories” of different jurisdictions. It is especially critical that we learn lessons that can improve the care, conditions, and outcomes of older adults and vulnerable populations such as those living in LTC (e.g., Armstrong, Armstrong, Choiniere, Lowndes, & Struthers, 2020). It is important that this work start now, both to track the policy and public health responses to COVID-19 that change by the day and to understand the experiences of the affected older adults and their families during and after the pandemic. It is also important that this work is holistic and considers the health and social consequences of COVID-19, as well as the pre-existing weaknesses of the system in supporting the physical, mental, and social well-being of older Canadians.

## Conclusion

In the preceding sections of this joint CAG/ACG and *CJA*/*RCV* statement, we have discussed just some of the key issues and opportunities pertaining to gerontology for consideration in the response to COVID-19. Although an extensive list of topics is beyond the scope of this statement, these examples nonetheless highlight the importance of interdisciplinary approaches during this time of significant personal and societal change. As we have demonstrated within and across the sections, COVID-19 gerontological research and applied practice must be comprehensive. There is value added when multiple perspectives and intersections of different disciplines are realized, rather than relying on one discipline alone.

Just as a single discipline will not be able to explain this phenomenon fully, neither will one person – collaboration is needed. We advocate for the development of multidisciplinary teams that bring together and bridge different areas of expertise as well as for multiple methods of information gathering, data collection, evaluation, and reporting. With these combined assets, we can work together on interdisciplinary topics to critically and comprehensively assess how older adults and the aging population are impacted by COVID-19. We encourage each of you – researchers, professionals, organizational representatives, students, caregivers, and older adults – to return to your respective disciplines, professions, and communities and to consider how you can forge and contribute to multidisciplinary teams. When ready to disseminate your work, we invite you to submit to a forthcoming call for papers on COVID-19 in the *Canadian Journal on Aging/La revue canadienne du vieillissement.* In the meantime, we hope this statement provides a level of focus and direction as we work together to examine COVID-19 from multiple gerontological points of view.
